# 2D vs. 3D Ultrasound Diagnosis of Pediatric Supracondylar Fractures

**DOI:** 10.3390/children10111766

**Published:** 2023-10-31

**Authors:** Jessica Knight, Fatima Alves-Pereira, Christopher E. Keen, Jacob L. Jaremko

**Affiliations:** 1 Department of Radiology and Diagnostic Imaging, Walter C. Mackenzie Health Sciences Center, University of Alberta, 8440-112 Street, Edmonton, AB T6G 2B7, Canada; jknight@ualberta.ca (J.K.); alvesper@ualberta.ca (F.A.-P.); 2 Department of Biomedical Engineering, Donadeo Innovation Center for Engineering, University of Alberta, 116 Street NW, Edmonton, AB T6G 2E1, Canada; ckeen@ualberta.ca

**Keywords:** POCUS, 3D ultrasound, elbow, supracondylar, fracture, effusion, pediatric

## Abstract

Supracondylar fractures are common injuries in children. Diagnosis typically relies on radiography, which can involve long wait times in the ED, emits ionizing radiation, and can miss non-displaced fractures. Ultrasound (US) has the potential to be a safer, more convenient diagnostic tool, especially with new highly portable handheld 2D point of care US (POCUS). This study aimed to determine the reliability of 2D POCUS for the detection of supracondylar fractures and elbow joint effusions, to contrast the accuracy of 2D POCUS vs. 3DUS vs. radiographs, and to determine whether blinded image interpretation could produce similar results to non-blinded real-time imaging. Fifty-seven children were scanned with 2D POCUS and 3DUS on the affected elbow. US scans were then read by three blinded readers, and the results were compared to gold-standard radiographs. Compared to a gold standard of 30-day radiographic diagnosis, readers of 2D POCUS detected supracondylar fracture and effusion with sensitivities of 0.91 and 0.97, respectively, which were both higher than with 3DUS. Inter-rater reliability of fracture detection was moderate for 2D POCUS (k = 0.40) and 3DUS (k = 0.53). Consensus sensitivities, although high, were lower than reports from some non-blinded studies, indicating that clinical presentation serves as an important factor in detection rates. Our results from consensus US diagnosis support the validity of using 2D POCUS in children for supracondylar fracture and elbow effusion diagnosis.

## 1. Introduction

Elbow fractures are common in the pediatric population, representing 3–7% of all fractures in children [1]. Of these, more than half are supracondylar fractures. Supracondylar fractures are most frequently diagnosed in children aged 5 to 10 years and show an equal distribution among girls and boys [1,2]. Displaced supracondylar fractures can be associated with significant complications such as brachial artery injury in ~10% of patients and median nerve dysfunction (particularly the anterior interosseous branch) in as many as 50% of patients [2]. The injury classically results from a fall on an outstretched hand and arm, which leads to hyperextension at the elbow, causing the olecranon to be driven into the olecranon fossa, resulting in a fracture [2]. 

The diagnosis of supracondylar fractures typically involves clinical examination followed by standard anteroposterior and mediolateral radiographs when appropriate [3,4]. Upon presentation, the child may complain of regional pain and swelling over the elbow, and careful inspection is required to assess for signs of neurovascular injury, such as puckering of the skin in the antecubital fossa or an abnormal neurological exam [3].

The supracondylar humerus, which is the thinnest part of the bone and most prone to fracture, is subject to silhouetting on lateral radiographs due to the projection of multiple bony prominences over a single area. Because of this, non-displaced supracondylar fractures are frequently occult on initial radiographs, requiring the radiologist to rely on the presence or absence of a joint effusion to determine their index of suspicion [5]. In these instances, patients are often treated with cast immobilization with interval repeat imaging [5]. Though this approach can be helpful in preventing complications of missed fractures, inevitably, some children are immobilized unnecessarily, and their daily activities are affected [5]. Another limitation is that children often have to wait for long periods of time in the emergency departments (ED) and often move between departments to undergo their radiographs as a result of the increased demand for healthcare services and the immobility of diagnostic imaging services [6]. Furthermore, radiographic images may not be interpreted promptly, compounding the in-hospital wait times families experience before children are discharged in the absence of fracture or receive care for their fracture [6]. 

Recently, the use of point-of-care ultrasound (POCUS) in the setting of musculoskeletal pain has increased [7]. At the elbow, POCUS allows for rapid bedside assessment and is effective in the identification of cortical disruption and periosteal fluid associated with fractures [8]. The ultrasound beam can directly observe the portion of the supracondylar fossa, which is hidden on lateral radiographs. Ultrasounds are also highly sensitive in the detection of joint effusion, with the added benefit of being able to evaluate complex effusions indicative of lipohemarthrosis, another positive indicator of fracture [5,8]. Recent studies have demonstrated that POCUS performed by pediatric emergency doctors with appropriate training in musculoskeletal sonography can have a high sensitivity and specificity for supracondylar fractures compared to radiographs [8,9,10]. Additionally, highly portable handheld POCUS probes that can easily be connected to a portable smartphone or tablet device have become readily available, which has led to a growing opportunity for the adoption of POCUS in the ED setting and has made POCUS accessible to physicians or healthcare professionals of varying skill levels [6]. One major drawback to ultrasound is that it is entirely user-dependent, and the required training and repeated exposure that is necessary for medical professionals to use it effectively and interpret the findings is not broadly accessible, limiting its utility [6]. 

The aims of our study were to (1) evaluate the feasibility, accuracy, and reliability of new, highly portable 2D POCUS in detecting supracondylar humeral fractures and joint effusions; (2) determine whether 2D POCUS performs comparably to high-resolution 3DUS performed on traditional full-sized US machines; and (3) determine whether blinded expert interpretation of supracondylar US images collected by non-expert scanners and read by remote experts produces similar results to un-blinded ED provider diagnostic accuracy, as demonstrated by Eckert et al. [8].

## 2. Materials and Methods

### 2.1. Study Design

This was a prospective diagnostic study performed at the Stollery Children’s Hospital in Edmonton, Alberta, Canada. It was approved by the University of Alberta Health Research Ethics Board—Biomedical Panel (Pro00077093) on 28 October 2020.

### 2.2. Clinical Protocol

Children ≤ 17 years old who presented with upper extremity trauma were identified at the triage desk of the Stollery Children’s Hospital ED. Only children with elbow, distal humerus, or proximal forearm tenderness following trauma were included in this study. Children with open wounds, compound fractures, or an existing cast over the elbow were excluded. Any children who were unable to tolerate the exam were also excluded. Each child and their legal guardian provided informed consent prior to participation in the study. Prior to being assessed by an ED physician, both 2D POCUS and 3DUS images were collected in the ED waiting room. No information regarding the US findings was communicated to the physician, who then provided standard clinical assessment and management to each child. 

### 2.3. Training

2D POCUS and 3DUS images were collected by a medical student with a diploma in diagnostic medical sonography and an undergraduate biomedical engineering student. Each student attended a 1 h training session, which included a demonstration of how to operate the US machines and a discussion of the image protocol, normal anatomy, and possible sonographic findings associated with supracondylar fractures. The session was provided by a pediatric emergency physician and a pediatric musculoskeletal (MSK) radiologist. Asynchronous, blinded readers did not receive any formal training. 

### 2.4. Ultrasound

To collect the ultrasound images, each child was asked to sit in a chair and place their symptomatic arm on a table in front of them. 3DUS images were acquired with a Philips IU22 machine and a 13 MHz 13VL5 probe (Philips, Amsterdam, NL, The Netherlands). The 2D POCUS images were acquired with an L5-12 MHz Philips Lumify probe (Philips, Amsterdam, NL, The Netherlands) and a tablet computer using Android OS (Alphabet Inc., Mountain View, CA, USA).

3DUS and 2D POCUS images consisted of a video sweep (cine) through (1) the dorsal aspect of the distal humerus over the olecranon fossa and (2) the volar aspect of the distal humerus over the coronoid and radial fossa. The dorsal images were collected with the child’s elbow flexed to 90 degrees and the volar images with the child’s elbow extended as close to 180 degrees as they could manage. Examination with US resulted in 2 3DUS sweeps and 2 2D POCUS sweeps, for a total of 4 US sweeps from each child. 

### 2.5. Radiographs

**Figure 1:**
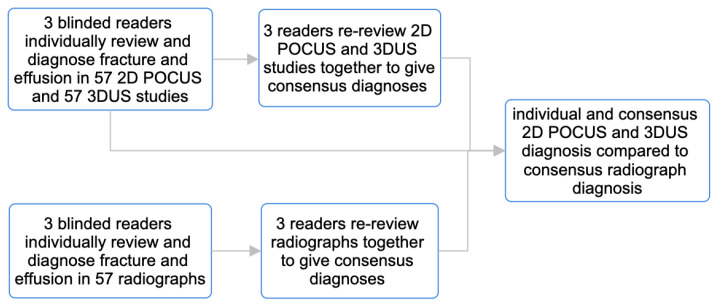
Protocol that was followed for radiograph and US image analysis to establish gold standard radiograph diagnosis, individual US diagnosis and consensus US diagnosis.

The majority of children who received a US also received routine radiographs of the same elbow during their ED visit, which were ordered by the ED physician to help direct clinical management. After a 30-day period following each child’s ED visit, we performed a chart review to discern whether any occult fractures may have been missed on initial radiographs. The radiographs that were collected were then anonymized, randomized, and interpreted by 3 blinded readers, including a dual fellowship-trained pediatric MSK radiologist (expert 1), a pediatric radiology fellow (expert 2), and a medical student with a diploma in diagnostic medical sonography (intermediate) (Figure 1). The consensus diagnosis reached by the 3 readers was then used as the radiograph gold standard for this study.

### 2.6. Image Analysis

Only children who had both an US and radiographs performed of their affected elbow were ultimately included in this study, and those with US only were excluded. Similarly to the radiographs, all 2D POCUS and 3DUS images were randomized, anonymized, and individually interpreted by the same 3 blinded readers (expert 1, expert 2, and intermediate). The readers provided their diagnosis as either ‘supracondylar fracture present’ or ‘supracondylar fracture not present’ and also indicated either ‘effusion present’ or effusion not present’. Each individual’s diagnosis was then compared to the radiograph consensus gold standard. Once all individual diagnoses were complete, all 3 readers then re-reviewed all US images together to determine a consensus US diagnosis (Figure 1). Of note, US studies were only characterized as ‘positive’ for supracondylar fracture if cortical disruption was visible. We did not count complex effusions that were suspicious for fracture as true positives. All 3 readers remained blinded to correlative clinical information for the entirety of the analysis. 

### 2.7. Statistical Analysis

Sensitivity, specificity, positive predictive value (PPV), and negative predictive value (NPV) were calculated for pooled individual and consensus fracture and effusion detection using consensus radiograph diagnosis as the gold standard. Inter-rater reliability was quantified with Fleiss’ Kappa [11]. Statistical significance of pooled individual 2D POCUS and 3DUS diagnoses were evaluated with McNemar’s test [12]. All statistical calculations were performed using IBM SPSS Version 29.0.1.0 (171).

## 3. Results

**Figure 2:**
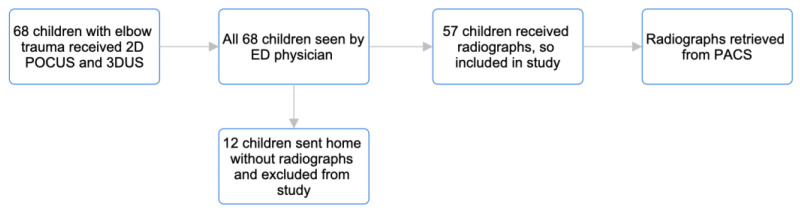
Pathway that was used for US and radiograph data collection by students performing scans at triage.

This study initially enrolled 68 children, but only 57 went on to receive both US and radiographs, which resulted in 11 children being excluded from the study. In total, we acquired 55 2D POCUS studies, 54 3DUS studies, and 218 individual US sweeps of symptomatic elbows (Figure 2). Secondary to technical issues or intolerance of the exam by the child, two children received only a 3D scan, and three children received only a 2D scan. There were nine scans where only the dorsal sweep could be obtained due to the child’s inability to straighten their elbow for adequate imaging of the volar surface.

Our results demonstrated variable detection rates of supracondylar fractures between the three readers for 2D POCUS and 3DUS, with moderate inter-rater reliability for both 2D POCUS (k = 0.40) and 3DUS (k = 0.53). Pooled sensitivity for fracture detection was 0.76 with a range of 0.64 to 0.91 for 2D POCUS and 0.64 with a range of 0.46 to 0.73 for 3DUS (Table 1). A consensus review of US images revealed 0.91 sensitivity for 2D POCUS and 0.82 for 3DUS fracture detection (Table 2). On the other hand, supracondylar fracture detection was more specific using 3DUS than it was with 2D POCUS (0.91 vs. 0.86 pooled and 0.95 and 0.86 via consensus, respectively, Table 1 and Table 2).

Effusion detection was more sensitive using 2D POCUS than it was with 3DUS (pooled sensitivity 0.97 and 0.93, respectively, Table 3), while 3DUS was more specific for effusion than 2D POCUS (0.76 vs. 0.67, respectively, Table 3).

Following a review of all the images, it became apparent that seven studies had fractures that were outside of our predetermined scan area for this study and thus were negative for supracondylar fracture, but a fracture in close proximity was missed. These cases were deemed true negatives for the purposes of this study.

## 4. Discussion

With the development of highly portable POCUS probes, POCUS-aided diagnosis of MSK injuries is undergoing increasing adoption in the ED setting [13]. In this study, we assessed the feasibility of utilizing 2D POCUS for the diagnosis of supracondylar humeral fractures and compared its efficacy to that of 3DUS. While the pooled data from individual readers showed variable results, the consensus reads were supportive of previous studies that have investigated the use of a US for supracondylar fracture detection and demonstrated its utility and feasibility [8,9,10]. Our study is unique because it examines the accuracy of highly portable 2D POCUS vs. high-resolution 3D US and tests the utility of having US scans performed by non-physicians with minimal US training as early as triage, with image interpretation occurring remotely by expert readers. It is important to note that ultrasound at triage would likely be most useful, serving as an initial investigation used to distinguish which children require assessment by an ED physician or further imaging with radiography, allowing those without fracture or effusion to avoid long wait times and radiation exposure.

**Figure 3:**
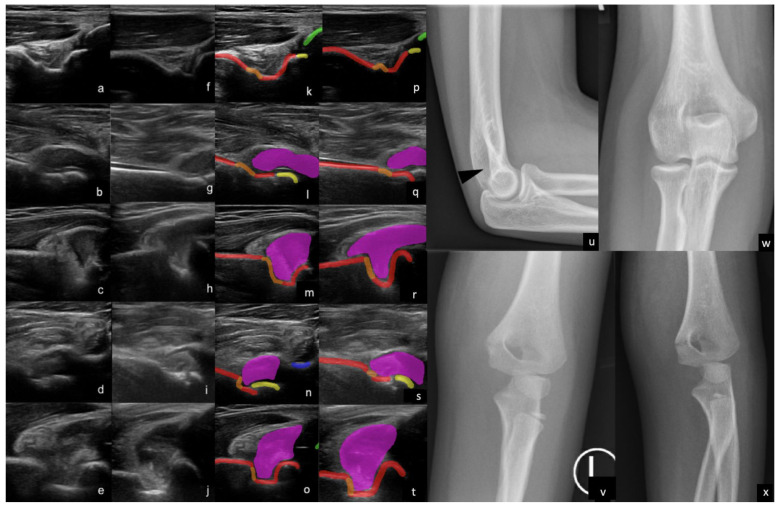
Examples of supracondylar fractures and elbow effusions with anatomy labels superimposed (red = humerus, orange = fracture, yellow = capitellum/trochlea, green = ulna, blue = radius, magenta = effusion). Buckle fracture on a dorsal 2D (**a**) and 3D (**f**) US images, with corresponding labels (**k**,**p**). Cortical step deformity, effusion, and fat pad elevation representing a fracture on a volar 2D (**b**) and 3D (**g**) US image, with corresponding labels (**l**,**q**). Sharp step of the olecranon fossa, effusion, and fat pad elevation suggestive of a supracondylar fracture on dorsal 2D (**c**) and 3D (**h**) US images, with corresponding labels (**m**,**r**). Subtle cortical interruption and effusion representing a minimally displaced fracture on volar 2D (**d**) and 3D (**i**) US image, with corresponding labels (**n**,**s**). Subtle cortical discontinuity in the superior aspect of the olecranon fossa representing the fracture on dorsal 2D (**e**) and 3D (**j**) US images, with corresponding labels (**o**,**t**). Radiographs (**u**,**w**) corresponding to the 2/3D US images (**a**,**f**,**k**) and (**p**). Note the subtle contour irregularity in the olecranon fossa cortex (black arrow head), which is suspicious for a buckle fracture and much more readily apparent on US. Radiographs (**v**,**x**) corresponding to (**b**,**g**,**l**) and (**q**) 2/3D US images demonstrating the fracture line. Note the marked soft tissue swelling. This patient was unable to flex their elbow; though an effusion is highly suspected, it is not actually demonstrated on radiographs, but can be easily observed on both volar and dorsal 2/3D US images.

We found that 2D POCUS was more sensitive than high-resolution 3DUS for both supracondylar fracture and elbow effusion detection when read by experienced readers. This may be because our 2D POCUS field of view was larger and more adaptable than the 3DUS field of view. The low specificity of supracondylar fracture and elbow joint effusion detection may relate to a limitation of the gold standard, potentially indicating that a US is more sensitive than radiographs in some cases and may even allow for the detection of some radio-occult fractures. As an example, there were five 2D POCUS cases where the US was arguably superior to radiographs in the detection of a fracture (i.e., the corresponding radiograph appeared negative, but ultrasound findings were clearly positive). Examples included the detection of subtle torus-like abnormalities in the cortex and subtle cortical disruptions and angulations in keeping with non-displaced fractures (Figure 3). Similarly, there were three 3DUS studies that were superior in the detection of non-displaced fractures, demonstrating the abnormalities described above (which were inconspicuous on the corresponding radiographs). One false-positive 2DUS study appeared to be caused by a relatively sharp edge of the olecranon/coronoid fossa, mimicking a fracture.

The results of this study also suggest that clinical suspicion and a physical exam may affect the sensitivity of POCUS for supracondylar fracture detection. Eckert et al. were able to detect supracondylar fractures with CUS with 1.00 sensitivity when un-blinded to clinical data [8], whereas our most sensitive reader was only able to achieve 0.91 sensitivity.

Kappa values showed moderate agreement between readers, with the sensitivity of fracture detection by 2D POCUS and 3DUS ranging from 0.64 to 0.91 and 0.46 to 0.73, respectively. Given the operator-dependent nature of a US, this variability is likely to increase even further with limited user experience, as it does in similar MSK applications of POCUS [6].

This was a limited single-center study with only 57 children included, which inherently creates some level of selection bias. It is unlikely that our small sample size was truly representative of the population, and it did not include all possible presentations of supracondylar fractures. In the future, we hope to confirm our findings with a larger study across multiple centers. Another limiting factor was that radiographs were used as our gold standard, as they are currently the imaging standard in clinical practice [14]; however, they are not perfect. For example, on radiographs that are positive for joint effusion but with no evidence of a clear fracture, there are occult elbow fractures in 17% to 50% of cases [15,16,17]. We were unable to employ a more sensitive gold standard in this study, such as MRI or CT, due to logistical difficulties and radiation dose, respectively. There were seven studies that had fractures outside of our predetermined scan area. In future research, we plan to expand our protocol to include more images, particularly more proximal views of the humerus, more distal views of the radius and ulna, and dedicated views of the medial and lateral epicondyles. It may also be useful to target a narrower age group for investigation in future studies as the anatomy of the elbow varies greatly between the ages of 0 and 17 and could have led to some difficulty for readers in determining expected normal anatomy, especially when blinded to all clinical data [18]. Similar to other MSK ultrasound applications, we expect that the elbow ultrasound data that we have collected will be highly amenable to real-time analysis and interpretation with AI [6]. In future research, we plan to investigate whether AI can decrease variability between readers and increase the accuracy and reproducibility of supracondylar fracture diagnosis using 2D POCUS.

## 5. Conclusions

We found that new, highly portable 2D POCUS was feasible for detecting supracondylar humeral fractures and elbow joint effusions and that it performs better than 3DUS on traditional US machines. Our data suggest that individual blinded radiologist/sonographer interpretation of US collected by non-experts produces inferior results for fracture detection when compared to real-time scanning and diagnosis by un-blinded expert ED providers; however, the sensitivity of fracture detection improved with consensus reads. These results confirm the intuitive conclusion that clinical suspicion can aid in the accuracy of supracondylar fracture detection.

## Figures and Tables

**Table 1:** Pooled sensitivities, specificities, PPV, and NPV of 2D POCUS vs. 3D US supracondylar humeral fractures detection by all 3 readers when compared to the radiograph gold standard.

Fracture Detection p = 0.035	Pooled Individual 2D POCUS Fracture Data (Range)	Pooled Individual 3DUS Fracture Data (Range)
Sensitivity	0.76 (0.64–0.91)	0.64 (0.46–0.73)
Specificity	0.86 (0.71–0.96)	0.91 (0.84–0.95)
PPV	0.65 (0.44–0.78)	0.68 (0.53–0.80)
NPV	0.94 (0.91–0.97)	0.91 (0.87–0.93)

**Table 2:** Consensus review sensitivities, specificities, PPV, and NPV of 2D POCUS and 3D US supracondylar humeral fracture detection when compared to the radiograph gold standard.

Fracture Consensus Review p = 0.063	2D POCUS (0.95 CI)	3D US (0.95 CI)
Sensitivity	0.91 (0.59 to 1.00)	0.82 (0.48 to 0.98)
Specificity	0.86 (0.73 to 0.94)	0.95 (0.84 to 0.99)
PPV	0.63 (0.44 to 0.78)	0.82 (0.53 to 0.95)
NPV	0.97 (0.85 to 1.00)	0.95 (0.85 to 0.99)

**Table 3:** Pooled sensitivities, specificities, PPV, and NPV of 2D POCUS vs. 3D US elbow effusion detection by all 3 readers when compared to the radiograph gold standard.

Effusion p = 0.019	Pooled Individual 2D POCUS Effusion Data (Range)	Pooled Individual 3DUS Effusion Data (Range)
Sensitivity	0.97 (0.96–1.00)	0.93 (0.92–0.96)
Specificity	0.66 (0.38–0.93)	0.76 (0.47–0.90)
PPV	0.75 (0.59–0.93)	0.78 (0.58–0.89)
NPV	0.97 (0.95–1.00)	0.92 (0.88–0.96)

## Data Availability

The data presented in this study are available upon request from the corresponding author. The data are not publicly available due to patient privacy requirements of clinical data.
